# Sedimentary hiatus causes abrupt decline and shifts in marine subsurface sediment microbial communities: a study from IODP Exp. 378 Site U1553 offshore southern New Zealand

**DOI:** 10.1093/femsle/fnag058

**Published:** 2026-05-14

**Authors:** Fumiaki Mori, Ann G Dunlea, Tatsuhiko Hoshino, Erika Tanaka, Kazutaka Yasukawa, Takeshi Terada, Yuki Morono

**Affiliations:** Kochi Institute for Core Sample Research, Japan Agency for Marine-Earth Science and Technology (JAMSTEC), Monobe, Nankoku, Kochi 783–8502, Japan; Marine Core Research Institute, Kochi University, Monobe, Nankoku, Kochi 783-8502, Japan; Marine Chemistry and Geochemistry Department, Woods Hole Oceanographic Institution, Woods Hole, MA 02543, United States; Kochi Institute for Core Sample Research, Japan Agency for Marine-Earth Science and Technology (JAMSTEC), Monobe, Nankoku, Kochi 783–8502, Japan; Advanced Institute for Marine Ecosystem Change (WPI-AIMEC), Tohoku University, Sendai 980-8578, Japan; Advanced Institute for Marine Ecosystem Change (WPI-AIMEC), Japan Agency for Marine-Earth Science and Technology (JAMSTEC), Yokohama, Kanagawa 236-0001, Japan; Marine Core Research Institute, Kochi University, Monobe, Nankoku, Kochi 783-8502, Japan; Earth and Planetary Sciences Department, University of California, Santa Cruz, CA 95064, United States; Frontier Research Center for Energy and Resources, School of Engineering, The University of Tokyo, Bunkyo-ku, Tokyo 113-8656, Japan; Marine Works Japan Ltd., Yokosuka, Kanagawa 237-0063, Japan; Kochi Institute for Core Sample Research, Japan Agency for Marine-Earth Science and Technology (JAMSTEC), Monobe, Nankoku, Kochi 783–8502, Japan

**Keywords:** hiatus, marine subsurface sediment, microbial community, microbial cell abundance, Aerophobia, International Ocean Discovery Program

## Abstract

Hiatuses within marine sediment are discontinuities in stratigraphic sediment records, forming adjacent layers of different ages. To evaluate how such discontinuities relate to subsurface microbial distribution, we examined microbial cell abundance and 16S rRNA gene-based community structure along a sediment core from International Ocean Discovery Program Expedition 378 Site U1553 (offshore southern New Zealand). Cell abundances decreased logarithmically with depth but showed two step-like declines: a sharp drop across a major hiatus at ∼4 mbsf and a second decline at ∼21.5–25 mbsf that overlaps, within sampling resolution, a reported Mn–Fe transition in porewater chemistry. Across the major hiatus, community composition and alpha diversity shifted markedly, indicating a persistent imprint of depositional discontinuity on subseafloor communities. Within the interval where community data were obtained (1.5–21.5 mbsf), community variation was more strongly associated with sediment depth and age than with the measured porewater solutes. These observations highlight stratigraphic age discontinuities as important contextual features for interpreting subseafloor microbial biomass and community patterns.

## Teaser

This study highlights that geological gaps in sedimentary records, known as hiatuses, serve as critical contextual features for interpreting abrupt, drastic shifts in deep marine microbial communities.

## Introduction

Marine sediment covers 70% of Earth’s surface, beneath which there is an extensive microbial biosphere. Microbial abundance generally declines with depth below seafloor and age of sediment due to limited substrate availability, which is fundamentally constrained by surface ocean productivity and sedimentation rates (Kallmeyer et al. 2012, Parkes et al. 2014, Bar-On et al. 2018). Despite such extreme energy limitation, microbial cells and their activity have been identified at depths reaching 2.5 km sedimentary depth (Inagaki et al. 2015), and in sediment that are 101.5 million years old (Morono et al. 2020). Previous studies estimated that there are 2.9–5.4 × 10^29^ cells from micro-organisms under the seafloor (Kallmeyer et al. 2012, Parkes et al. 2014, Bar-On et al. 2018). This estimate has been refined over time by improving modeling approaches based on detailed data sets of horizontal and vertical cells distributions (Kallmeyer et al. 2012, Parkes et al. 2014, Bar-On et al. 2018). Although it is well known that the microbial cell abundances decrease logarithmically with sediment depth, environmental factors can also cause transient changes in cell abundances. Even in deep sediment layers, local geochemical conditions (e.g. sulfate-methane transition zone, organic-rich layers) can lead to localized increases in microbial abundance (Parkes et al. 2014). Additionally, microbial distribution can be fundamentally influenced by sedimentary hiatuses—discontinuities in stratigraphic sediment records, which form adjacent layers of significantly different ages. However, the extent to which these hiatuses impact microbial habitats remains unclear and requires focused investigations.

Hiatuses are reported from a number of deep-sea drilling sites worldwide. They can arise from seafloor erosion by strong bottom currents, biogenic carbonate dissolution associated with fluctuations in the carbonate compensation depth, and/or extremely low net sedimentation (condensed or “starved” intervals) (Dutkiewicz and Müller 2022). Additionally, tectonic activity can lead to hiatuses by decreasing sediment supplies through river rerouting (Marsaglia et al. 2011). In such settings, the long-term co-evolution of geochemical environments and microbial communities expected under continuous deposition may be altered. Consequently, adjacent layers separated by a major age gap may exhibit abrupt differences in microbial abundance and community structure, associated with severe energy limitation and/or shifts in redox history. Under such extreme energy shortages in the marine subsurface, micro-organisms are forced to adapt, and specific surviving groups are selected to form unique communities (Petro et al. 2017, Starnawski et al. 2017, Hoshino et al. 2020). Consequently, evaluating the impact of a significant age gap and the intensified energy limitation across a hiatus is crucial for understanding microbial distribution in the marine subsurface. To better understand these microbial dynamics, we characterize microbial abundance and community structure in subseafloor sediments and examine their relationship with sedimentary hiatuses.

## Materials and methods

### Sampling procedure

**Figure 1:**
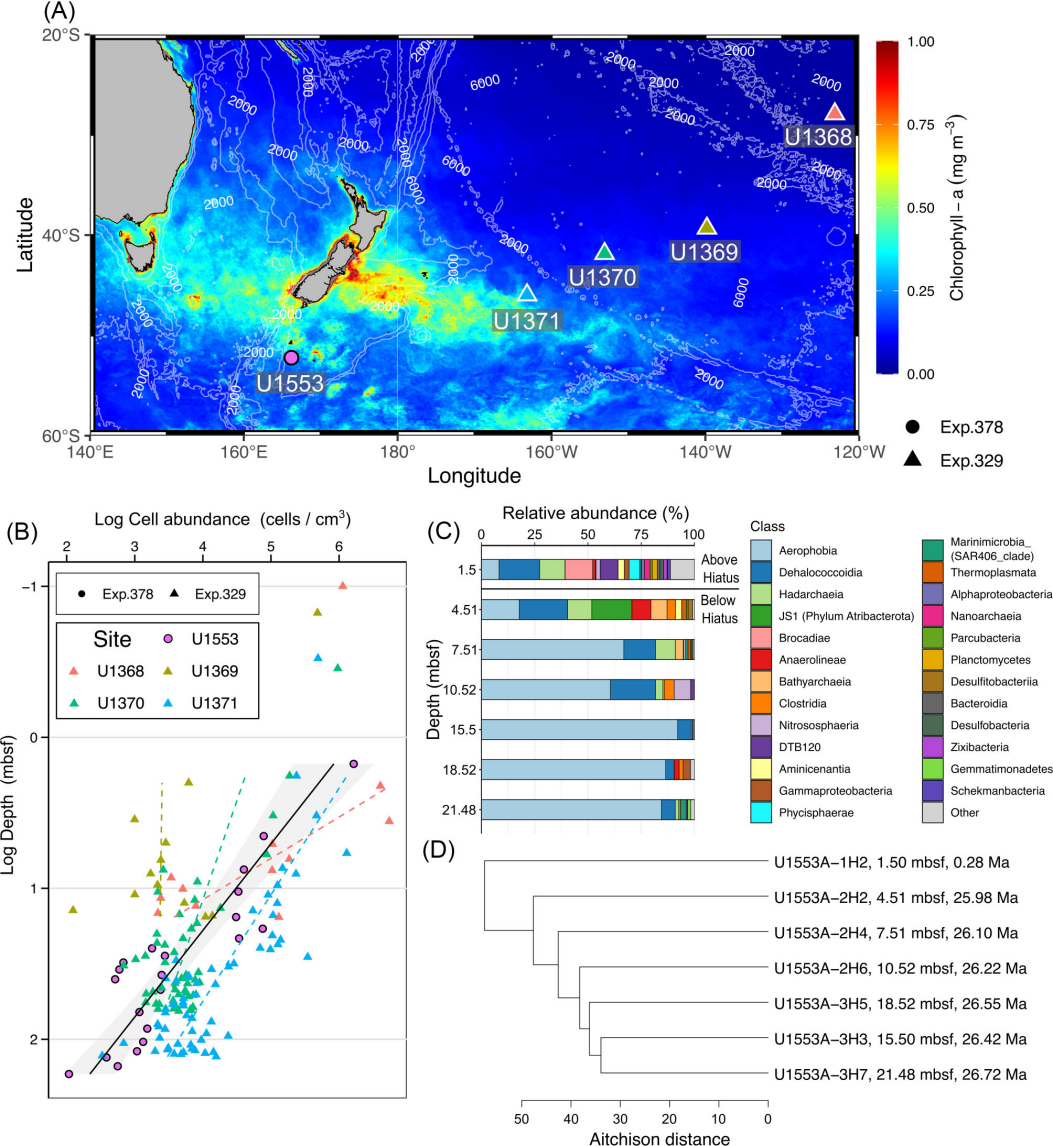
Microbial abundance and community composition at Site U1553. (A) Map showing the location of Site U1553 along with comparative sites in the South Pacific Gyre (U1368–U1371). The background shading indicates the annual average chlorophyll-*a* concentrations for 2019, as derived from the MODIS dataset. (B) Log cell concentrations versus log depth at Site U1553 (pink circle) and compared sites (triangle). The regression slope is depicted by a solid black line at Site U1553, while dashed lines represent the regression slopes for the other sites. The regression equations are as follows: Site U1368: Log cells = 7.9 − 3.7 Log depth (R² = 0.65); Site U1369: Log cells = 3.4 − 0.03 Log depth (R² = 0.0003); Site U1370: Log cells = 4.8 − 0.76 Log depth (R² = 0.35); Site U1371: Log cells = 6.5 − 1.4 Log depth (R² = 0.67); and Site U1553: Log cells = 6.2 − 1.74 Log depth (R² = 0.80). All regressions were statistically significant (*P* < 0.001) except that for Site U1369. (C) Relative abundance of microbial communities at the Class level, showing the top 25 most abundant classes, with the remaining taxa grouped as “Others”. (D) Hierarchical clustering based on Aitchison distance at the ASV level and was calculated using Ward’s method.

We investigated sediment cores collected during International Ocean Discovery Program (IODP) Expedition 378 at Site U1553 (52°13.43’S, 166°11.48’E, 1221.2 m water depth) located on the southern Campbell Plateau roughly 644 km (400 miles) south of New Zealand (Fig. 1A). During IODP Expedition 378 in January 2020, the drilling vessel JOIDES Resolution, recovered sediment from five holes (Holes A, B, C, D, and E) at Site U1553. While the Campbell Plateau is regionally known for the occurrence of polymetallic nodules (e.g. Carter 1989), such macroscopic nodules were not observed at Site U1553. Microbiological samples were taken from Hole U1553A (Röhl et al. 2022a). The sediment samples for microbial cell counts and community analysis were collected from surface to deeper layers, ranging from 1.5 to 169.8 m below seafloor (mbsf). To minimize potential contamination from drilling fluids and physical disturbances, microbiological subsamples were carefully extracted from the undisturbed inner portion of the sediment cores. For DNA extraction, we subsampled 5–10 cm^3^ of sediments with tip-cut syringes, which were subsequently placed in sterile bags and stored at –80°C. Approximately 2 cm^3^ of sediment samples for microbial cell counts were immersed in fixing solution (10% formalin and 3 × PBS) and then stored at 4°C.

Porewater geochemical data used in this study were retrieved from the previously published IODP Expedition 378 dataset (Röhl et al. 2022b). Geochemical samples were collected adjacent to the microbiological samples and analyzed during the expedition following the procedures described in Röhl et al. (2022a). Briefly, porewater was extracted from sediments using either a laboratory hydraulic press (Carver Inc., IN, USA) with subsequent filtration through 0.45 µm filters or Rhizon samplers (Rhizosphere Research Products, Wageningen, Netherlands). Dissolved Fe and Mn concentrations were determined by inductively coupled plasma optical emission spectrometry (ICP-OES; Agilent 5110, Agilent Technologies, CA, USA) using samples diluted with 2% ultrapure HNO₃ with dilution ratios of 1:10. The detection limit for Fe was reported as 0.2 µM, whereas a detection limit for Mn was not specified in Röhl et al. (2022a), although concentrations down to 0.13 µM were reported. Sulfate concentrations were measured by ion chromatography (850 Professional IC, Metrohm, Herisau, Switzerland), and ammonium concentrations were determined spectrophotometrically using an ultraviolet-visible spectrophotometer (Agilent Cary 100, Agilent Technologies, CA, USA) following the method of Solórzano (1969). Because microbiological and geochemical samples were collected from adjacent but not identical depths, a consistent offset of ∼5 cm exists between the datasets (microbiological samples being deeper). For example, geochemical data at 1.45 mbsf were paired with microbiological samples at 1.50 mbsf (Supplementary Table S1). To minimize uncertainties related to this offset, only dissolved porewater parameters were used for statistical analyses. In contrast to solid-phase parameters (e.g. total organic carbon), dissolved solutes diffuse through sediment pore spaces and typically exhibit smoother vertical gradients, making them more suitable for comparison across small depth offsets.

### Cell counts

To minimize the risk of contamination, we conducted all the microbiological sample handling (e.g. DNA extraction, cell separation, and cell-staining processes) under a laminar-flow clean air environment in a super-clean room of JAMSTEC-KOCHI (Morono et al. 2018). For sediment samples collected from the shallow layer (1.5 mbsf) that contained more than 10^6^ cells cm^–3^, we counted microbial cells using the method described by Morono et al. (2009), which does not involve any cell separation steps. For the samples with lower cell abundance, we employed a cell separation procedure following Morono et al. (2013), with the modifications described below. The use of cell separation procedure was necessary to bring the dynamic range of the cell counting down to tens of cells per cm^3^ sediment samples with > 80% recovery regardless of the type of the sediments (Morono et al. 2013). The 1 ml of fixed sediment slurry was mixed with 2.2 ml of 2.5% NaCl solution and added with 400 µl each of detergent mix (100 mM EDTA, 100 mM sodium pyrophosphate, 1% [v/v] Tween 80) and methanol, then vigorously shaken for 60 min at 500 rpm using a Shake Master (Bio Medical Science, Tokyo, Japan). After shaking, the sediment slurry was sonicated (Bioruptor UCD-250; Sonicbio, Kanagawa, Japan) in an ice bath for 20 cycles of 30 s at 200 W on and 30 s off. The processed slurry was then carefully layered onto a manually layered high-density cushion solution consisting of (from top) 2 ml of 30% [v/v] Nycodenz, 2 ml of 50% [v/v] Nycodenz, 2 ml of 80% [v/v] Nycodenz, and 2 ml of 67% [w/v] of sodium polytungstate. Samples were centrifuged at 10 000 × g for 1 h, after which the supernatant, including the high-density layer(s), was carefully removed and transferred to a separate vial. After removing high density layers, sediment pellet was mixed with 3 ml of NaCl solution and 400 µl each of detergent mix and methanol again, then cell separation steps were repeated. The cell-containing supernatants were pooled in the same vial. The cells in half of the pooled supernatant were trapped onto a 0.22 μm pore-sized black polycarbonate membrane (EMD Millipore, MA, USA), then stained with SYBR Green I, and counted by the fluorescence color-based discriminative cell enumeration method according to Morono et al. (2009). We observed across either a minimum of 10 fields of view (FOV) for the high-biomass samples exceeding 100 cells per 10 FOV, FOVs until total counts reaches 100, or entire membrane (>900 fields of view) to obtain as accurate as possible cell counts (Morono 2023).

### DNA extraction and 16S rRNA gene amplicon sequencing

DNA was extracted using the DNeasy PowerMax Soil Kit (Qiagen, Hilden, Germany), according to the manufacturer’s instructions with slight modifications. For the initial cell disruption step, we replaced the kit-provided bead with heat-combusted zirconia beads (φ0.1 mm and φ0.5 mm; 7.5 g each). Also, the disruption was performed using a Multi-beads shocker (Yasui Kikai, Osaka, Japan) at 2500 rpm for 180 s. All other steps followed the manufacturer’s protocol. For the microbial community composition (MCC) analysis, extracted DNA were amplified for Illumina MiSeq 16S rRNA gene amplicon sequencing. The PCR was conducted using MightyAmp DNA polymerase ver. 2 (Takara Bio, Shiga, Japan) with universal primers specific for the V4 regions of the 16S rRNA gene, 515F and 806R (Caporaso et al. 2011). The thermal cycling conditions consisted of an initial denaturation at 98°C for 2 min, followed by 31 or 34 cycles (determined based on preliminary monitoring of amplification curves) of denaturation at 98°C for 10 s, annealing at 55°C for 15 s, and extension at 68°C for 20 s. After gel electrophoresis, we excised the appropriate size of the band and purified DNA using the NucleoSpin Gel and PCR Clean-up kit (Takara Bio, Shiga, Japan). The second PCR was then conducted to index each of PCR product using KAPA HiFi HotStart ReadyMix (KAPA Biosystems, MA, USA). The cycling conditions were initial denaturation at 95°C for 2 min, followed by nine cycles of denaturation at 98°C for 20 s, annealing at 60°C for 15 s, and extension at 72°C for 30 s. After amplification, the second PCR products were purified twice using Agencourt AMPure XP beads (Beckman Coulter, CA, USA) for ensuring removal of small fragments. Subsequently, all samples were sent to the Bioengineering Lab. Co., Ltd. (Kanagawa, Japan) for sequencing on the Illumina MiSeq platform with the MiSeq Reagent Kit v3 (2 × 300 bp, Illumina, CA, USA). In addition to the environmental samples, we prepared the negative control (blank) samples at the step starting from the first PCR and processed under the identical protocol and reagents to monitor potential laboratory and reagent-derived contamination.

Raw MiSeq data were analyzed using Qiime2 (v2022.2; Bolyen et al. 2019) as described in Wakamatsu et al. (2021). In brief, the primer sequences were trimmed using the trim-paired function of the cutadapt Qiime2 plugin (Martin 2011). Following primer sequence removal, the denoise-paired function of dada2 plugin (Callahan et al. 2016) was used for quality control, including chimera removal, and amplicon sequence variants (ASV) were determined. Finally, the ASV were classified using pre-trained Naive Bayes classifier based on the SILVA 138 SSU Ref NR 99 database (Quast et al. 2013) and the q2-feature-classifier plugin. To minimize potential bias from contamination during sampling and laboratory processes, a three-step stringent manual curation was applied to the resulting ASV table. First, ASVs detected in the negative control (PCR blank) were entirely removed. Second, specific taxa belonging to orders identified as ubiquitous contaminants in scientific ocean drilling (e.g. *Staphylococcales*, including *Staphylococcus*; and *Pseudomonadales*, including *Acinetobacter*) were removed from the dataset, following the criteria established by Labonté et al. (2025). Finally, specific genera *Thermus, Deinococcus* (Salter et al. 2014), and *Legionella* (Shen et al. 2006) were excluded based on its known presence in commercial reagents and laboratory water systems. A list of the excluded taxa is provided in Supplementary Table S2. The results were imported into the R statistical environment (v4.0.4; R Core Team 2020) by using qiime2R packages (Bisanz 2018). For visualization and statistical test, the phyloseq (McMurdie and Holmes 2013), ggplot2 (Wickham 2016), and microViz (Barnett 2021) R packages were used. To mitigate compositional bias, the ASV table was centered log-ratio (CLR) transformed, with zero values handled by adding a pseudocount of half the minimum abundance. Based on the Aitchison distance (i.e. Euclidean distance of CLR-transformed data; Gloor et al. 2017), we performed BIOENV (Clark 1993) and Mantel tests (Mantel 1967) using the vegan package (Oksanen et al. 2019) in R to assess the effects of environmental parameters on microbial community shifts. The Aitchison distance was used to construct hierarchical clustering dendrograms and perform Principal Component Analysis (PCA), which were visualized using the ggdendro (de Vries and Ripley 2024) and microViz packages (Barnett 2021), respectively. For alpha diversity analysis, the ASV table was first rarefied to an even depth of 8550 reads per sample. Rarefaction curves confirmed that this sequencing depth was sufficient to capture the majority of the microbial diversity across all samples (Supplementary Fig. S1). Based on this rarefied table, observed richness and the Shannon index were calculated using the phyloseq package (McMurdie and Holmes 2013).

## Results and discussions

**Figure 2:**
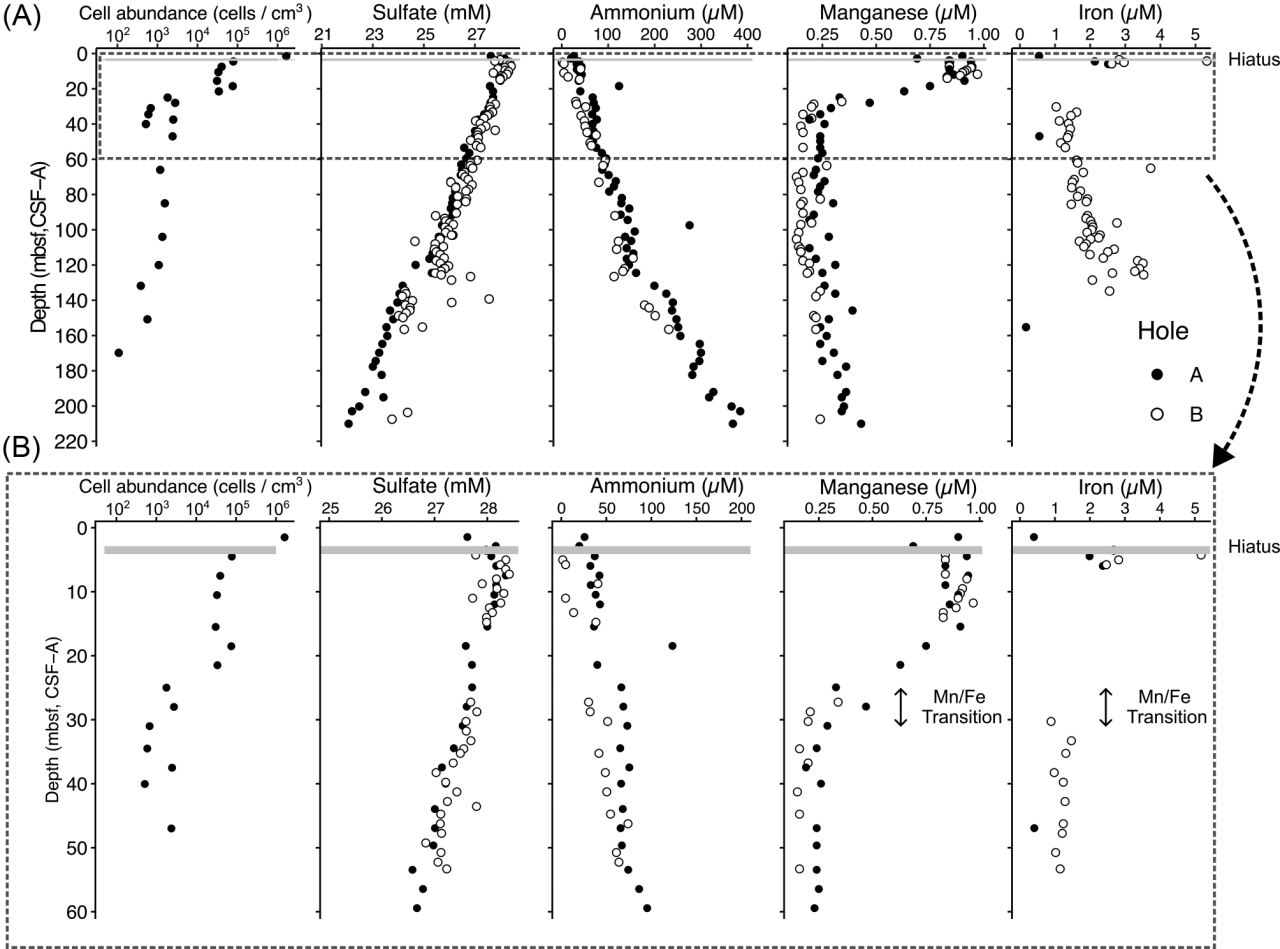
Cell abundance and porewater geochemical parameters (SO_4_^2–^, NH_4_^+^, Mn, Fe) profiles from Hole U1553A and B. (A) Full-core profiles illustrating the overall trends from 0 to 220 mbsf. (B) Magnified view of the upper 0–60 mbsf interval, highlighting the detailed geochemical and biological transitions. The gray line indicates the sedimentary depth of the major hiatus. All these porewater geochemistry datasets were retrieved from the previously published IODP Expedition 378 dataset (Röhl et al. 2022b).

A major hiatus was inferred at ∼4 mbsf by the calcareous nannofossil biostratigraphy, which confirmed upper Pleistocene taxa (0.017–2.6 Ma) above the hiatus (4 mbsf) and upper Oligocene (23–28Ma) below it (Röhl et al. 2022c). Cell abundances decreased logarithmically with depth overall, while two abrupt declines were observed. The first abrupt decline occurred across the hiatus at ∼4 mbsf. The shallowest sample at 1.50 mbsf (0.28 Ma) had 1.6 × 10^6^ cells cm^–3^ while cell abundances below the hiatus ranged from 3.0–7.8 × 10^4^ cells cm^–3^ between 4.51 and 21.48 mbsf (Figs. 1B, 2A, 2B). The second abrupt decline was observed between 21.48 and 24.99 mbsf (26.7 and 26.9 Ma, respectively), cell abundances decreased an order of magnitude to around 0.3–2.7 × 10^3^ cells cm^–3^ and continued to decline with depth to as low as ∼100 cells cm^–3^ in the deepest sample taken at ∼169.81 mbsf (33.1 Ma). The first decline coincided with the hiatus, consistent with a link between stratigraphic discontinuity and microbial abundance. The second abrupt decline lacks such a direct stratigraphic explanation. As detailed below in the porewater geochemical context (Fig. 2A and B), this interval overlaps, within our sampling depth resolution, a Mn–Fe redox transition (∼24–30 mbsf).

To provide a global context for the microbial distribution trends at Site U1553, we compared the cell abundances with data from IODP drill sites ranging from the center to the edge of the ultra-oligotrophic South Pacific Gyre (SPG) (Expedition 329; Sites U1368–U1371; Fig. 1A). The SPG sites have some of the lowest known microbial abundance in the global ocean sediments (D’Hondt et al. 2015). Three of the four sites are located in the SPG (U1368–U1370), while one site (U1371) is located in a more biologically productive area (D’Hondt et al. 2015). In general, microbial abundances in marine subsurface strongly correlate with mean sedimentation rate and distance from land (Kallmeyer et al. 2012). Although Site U1553, located ∼644 km from New Zealand, showed mean sedimentation rates of 1.1–3.3 cm/kyr (Röhl et al. 2022c), substantially higher than those reported for the SPG (0.008–0.178 cm/kyr; D’Hondt et al. 2009), cell abundance at Site U1553 declined from 1.51 mbsf to below the hiatus layer at 4.51 mbsf, reaching levels comparable to those at the ultra-oligotrophic SPG Sites U1369–U1370 (Fig. 1B). Using previously reported sedimentation rates, the estimated age of the 4.51 mbsf layer in the ultra-oligotrophic SPG would range from 12–14 Ma at Site U1368 (Alvarez Zarikian 2015), 6–20 Ma at Site U1369 (Dunlea et al. 2015, Tegler et al. 2025), 2–8 Ma at Site U1370 (Dunlea et al. 2015, Tanaka et al. 2024, Tegler et al. 2025), < 1 Ma at Site U1371 (Cortese and Alvarez Zarikian 2015, Suto and Uramoto 2015), which is on the same order of magnitude as the 26 Ma age at Site U1553 at the same depth (4.51 mbsf, Supplementary Table S1). Because microbial cell abundance in marine sediments is known to be strictly constrained by sediment age, the comparably low cell abundances observed at Site U1553 and the ultra-oligotrophic SPG sites can be attributed to their similarly old ages at this depth. Although Site U1553 is located closer to land and has a higher sedimentation rate than the ultra-oligotrophic SPG, shallow sediments at Site U1553 contain relatively older sediment due to the presence of a hiatus. These observations are consistent with the idea that the shallow sediments at Site U1553 are unusually old because of the stratigraphic discontinuity. Such old sediment age at shallow depth is likely associated with strong energy limitation, which may help explain the comparably low cell abundances observed at Site U1553 and the ultra-oligotrophic SPG sites.

MCC analysis was conducted for the seven sediment samples ranging from 1.50 to 21.48 mbsf where cell concentration was above 10^4^ cells cm^–3^ and preliminary PCR amplification succeeded. A total of 487 amplicon sequence variants (ASV) were recovered (Supplementary Table S3). Analyzing MCC in deep-subsurface sediments poses inherent challenges due to extremely low biomass and limited DNA yields. To obtain sufficient amplicons from these samples, we conducted PCR amplifications with cycles exceeding 30. Although we acknowledge that such intensified amplification may introduce artificial taxonomic biases and increase the potential for amplifying contaminating sequences, >30 cycles of PCR amplifications were necessary to yield analyzable amounts of PCR products even we minimized the number of cycles as much as possible. To address the contamination risk, we applied stringent manual curation and removal of non-indigenous sequences based on both the criteria for ocean drilling environments (Labonté et al. 2025) and known laboratory contaminants (Shen et al. 2006, Salter et al. 2014). Specifically, ASVs that matched those from negative controls or were identified as potential contaminants were excluded from further analysis (Supplementary Tables S2 and S3). These combined experimental and bioinformatic efforts ensure that the resulting 487 recovered ASVs represent a high-confidence and robust profile of the indigenous MCC in this extreme environment. The most dominant Class was *Aerophobia* accounting for 59.6% (8.3%–92.1%), followed by *Dehalococcoidia* (Mean 13.5%; 4.0%–22.7%), *Hadarchaeia* (Mean 5.5%; 0%–11.9%), JS1 (Mean 2.7%; 0%–18.8%), and *Brocadiae* (Mean 1.9%; 0%–13.0%) (Fig. 1C). Notably, the shallowest sediment (1.50 mbsf) exhibited a unique community signature, including the exclusive detection of an anammox bacterium *Candidatus* Scalindua (12.6% of the family Scalinduaceae within the family-level community). This lineage is known for its relatively high oxygen tolerance (Okabe et al. 2023). Furthermore, specific clades reported to dominate in the oxic subseafloor (Vuillemin et al. 2020a), such as the *Dehalococcoidia* clade SAR202 (Mean 0.7%; 0%–3.5% within the order-level community) and S085 (Mean 0.6%; 0%–1.4% within the order-level community, Supplementary Fig. S2), were detected intermittently across the analyzed layers. The presence of these groups suggests the possibility that suboxic levels of oxygen might penetrate into the layers where the MCC was analyzed. However, because dissolved oxygen concentrations were not measured at Site U1553, the exact redox conditions throughout the core remain unresolved.

We evaluated the potential influence of available porewater geochemical parameters (e.g. sulfate, ammonium, and manganese concentrations) on the overall community structure, but none showed significant correlations. Instead, successive shifts in MCC were observed throughout the core along with increasing sedimentary logarithmic depth (Mantel test, r = 0.83, *P* < 0.01) and age (Mantel test, r = 0.71, *P* < 0.05). Consistent with this age-related trend, the MCCs at 1.50 and 4.51 mbsf, separated by a hiatus with an age gap of over 20 Ma, formed distinct clusters (Fig. 1D). This pronounced structural shift was further supported by principal component analysis based on Aitchison distance, which clearly separated the communities above and below the hiatus along the primary axis (Supplementary Fig. S3). Coinciding with this compositional shift, community diversity, including the Shannon index and observed ASVs, also decreased markedly across the hiatus (Supplementary Fig. S4). Together, these observations indicate that the abrupt age discontinuity at ∼4 mbsf is strongly associated with a shift in subseafloor microbial community structure. One plausible explanation is that, across the hiatus, much older sediment occurs at relatively shallow depth, where bioavailable energy substrates are likely more strongly depleted than would be expected based on depth alone. Reduced sediment accumulation and/or prolonged near-surface exposure prior to final burial may have contributed to stronger depletion of labile organic matter, thereby favoring micro-organisms adapted to highly recalcitrant substrates and severe energy limitation (Bradley et al. 2020). Our data are consistent with this interpretation, although the depositional mechanism responsible for the hiatus (e.g. erosion, non-deposition, or a combination of both) cannot be resolved from the present dataset. In this study, we observed a sharp increase in several putatively fermentative bacterial group (e.g. JS1, *Anaerolinae*, and *Aerophobia*) below the hiatus at 4.51 mbsf (Fig. 1C). In particular, JS1 a lineage within the *Atribacterota* phylum that is commonly reported from energy-limited marine subsurface sediments (Hoshino et al. 2020, Vuillemin et al. 2020b), increased from 0.08% at 1.50 mbsf to 18.8% at 4.51 mbsf. This pattern is therefore consistent with adaptation of the MCC to increasingly energy-limited conditions below the hiatus, rather than depth alone.

**Figure 3:**
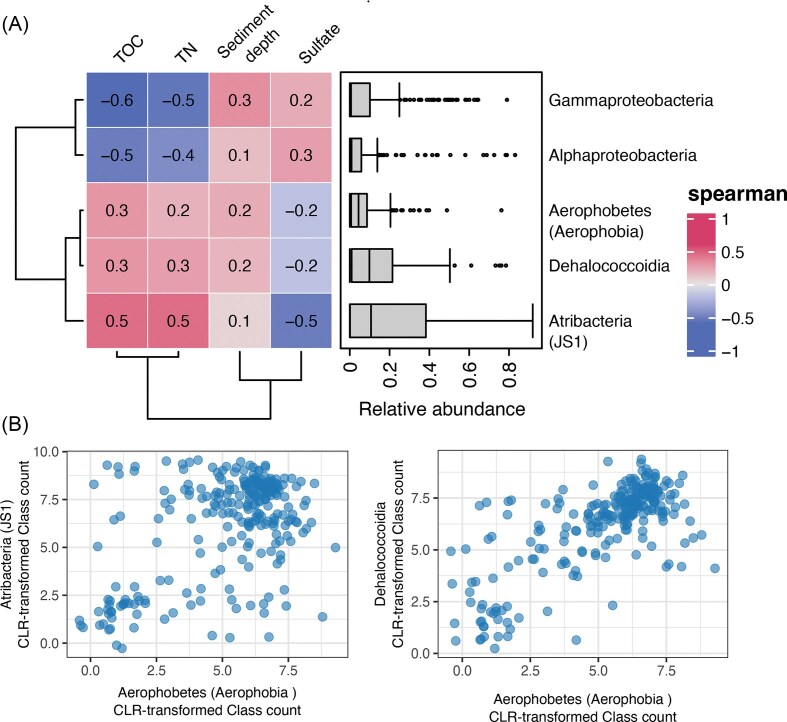
Correlation of *Aerophobia* with environmental parameters and other potential fermenters in the global marine subsurface microbial community composition dataset from Hoshino et al. (2020). (A) Correlation heatmap showing relationships between the top five class-level groups and environmental parameters: sediment depth, sulfate, total organic carbon (TOC), and total nitrogen (TN) (N = 253; samples lacking the geochemical data were excluded). To reduce compositional bias, class counts were transformed using the centered log-ratio (CLR) method (Gloor et al. 2017) prior to correlation analysis. Correlation coefficients are indicated within each cell. Boxplots represent the relative abundance of each class within the dataset. All environmental and geochemical parameters, including TOC, are derived from the global dataset Hoshino et al. (2020). (B) Relationships between *Aerophobia* and other predominant potential fermenter groups (JS1 and *Dehalococcoidia*). CLR-transformed class counts were also used in this analysis. Samples in which either of the two classes shown in the scatterplots was absent were excluded to avoid zero-value bias during CLR transformation (N = 251). Note that the dataset from Hoshino et al. (2020) was classified based on the SILVA 132 SSU database; therefore, some class-level names differ from those in this study using the updated SILVA 138 taxonomy (e.g. *Aerophobetes* corresponds to *Aerophobia*, and *Atribacteria* corresponds to JS1).

The hierarchical clustering analysis of the MCC at Site U1553 showed that MCC in sediments deeper than 7.51 mbsf formed a distinct cluster, which is characterized by the high abundance of *Aerophobia* (Fig. 1C and D). The relative abundance of *Aerophobia* shows a drastic increase from 17.8% at the 4.51 mbsf to 66.9% at the 7.51 mbsf and peaked at 15.50 mbsf showing 92.1%. While *Aerophobia* has been reported to be universally dominant in marine subsurface (Hoshino et al. 2020), environmental parameters affecting their abundance have not been intensively discussed and their distribution patterns remain poorly constrained. Although there is no representative isolates, previous studies using metagenome-assembled genomes have provided genomic evidence that *Aerophobia* can produce acetate via the Wood–Ljungdahl pathway and conduct fermentation (Wang et al. 2016, Dong et al. 2019). The remarkably high dominance of potentially acetogenic, fermentative *Aerophobia* observed in this study appear to exceed previously reported relative dominance in subsurface environments. In a global census of MCC in the marine subsurface (Hoshino et al. 2020), identified a few sites with exceptionally high *Aerophobia* dominance, reaching up to 76% of relative abundance. This aligns with previous findings, which further suggest that homoacetogens and fermenters including *Aerophobia* may play a crucial role in organic carbon cycling in the marine deep biosphere (Vuillemin et al. 2020b, Wasmund et al. 2014). To better understand *Aerophobia* distribution trends, we analyzed the global census MCC dataset from Hoshino et al. (2020). Across the global dataset, we did not identify strong monotonic relationships between Aerophobia relative abundance and the examined variables (TOC, TN, sulfate, or sediment depth) (Fig. 3). Aerophobia showed positive correlations with JS1 (ρ = 0.26, *P* < 0.001, N = 251) and *Dehalococcoidia* (ρ = 0.62, *P* < 0.001, N = 251). These correlations, however, do not necessarily reflect direct ecological interactions. Rather, they may indicate shared environmental preferences, particularly for necromass utilization in substrate-limited sediments. To minimize direct competition under severe substrate limitation, these clades likely employ niche partitioning by targeting distinct organic compounds as electron donors—for instance, JS1 potentially fermenting sugars and Dehalococcoidia potentially utilizing fatty acids, as inferred from genomic evidence ( Wasmund et al. 2014, Vuillemin et al. 2020b).

For geochemical context, we considered porewater profiles of sulfate, ammonium, Mn, and Fe (Fig. 2). Dissolved Mn remained > 0.6 µM from 0–21.43 mbsf and decreased to < 0.5 µM below ∼24 mbsf, whereas dissolved Fe increased below ∼24 mbsf and the Mn–Fe transition was completed by ∼30 mbsf. These profiles are consistent with redox zonation and/or changes in solid-phase Mn–Fe pools, but the present dataset does not allow us to distinguish among these mechanisms. We therefore avoid attributing the Mn and Fe profiles to active microbial processes alone.

Independent of the Mn–Fe profiles, porewater sulfate decreases gently with depth and ammonium increases approximately linearly, consistent with diffusive porewater gradients superimposed on low net reaction rates. Previous work at Site U1553 also reported solid-phase (e.g. presence of pyrite) and isotopic (i.e. dissolved sulfate δ^34^S and δ^18^O) evidence consistent with sulfate reduction (Reis et al. 2023). Within the depth interval where community data were obtained (1.5–21.5 mbsf), none of the measured porewater parameters showed significant correlations with overall community structure, whereas community composition tracked sediment depth and age more closely. The second step-like decline in cell abundance (21.5–25 mbsf) overlaps, within our sampling resolution, the interval of the Mn–Fe transition. However, because community data are not available below 21.5 mbsf, we cannot determine whether a corresponding community shift occurs across that deeper interval. Taken together, these observations suggest that long-term energy limitation associated with sediment age is a stronger driver of the observed community structure than the present-day porewater gradients measured here, while the mechanism behind the second biomass decline remains non-unique.

## Conclusion

In this study, we evaluated the microbial distribution and community composition in the marine subsurface affected by hiatuses at IODP Expedition 378 Site U1553, located south of New Zealand. Microbial cell counts were confirmed to decrease logarithmically with sedimentary depth, but abrupt declines in cell abundance were observed at the hiatus, with corresponding changes in microbial communities. These results indicate that major stratigraphic age discontinuities are important contextual features for understanding subseafloor microbial abundance and community structure. Future work integrating microbial analyses with finer-scale stratigraphic and solid-phase geochemical constraints will be needed to resolve the mechanisms underlying these patterns.

## Supplementary Material

fnag058_Supplemental_Files

## Data Availability

DNA sequences are available in the DDBJ Sequence Read Archive: DRA022533 (DRX729103-DRX729110). Porewater geochemistry datasets used in this study are available from the Laboratory Information Management System database (http://web.iodp.tamu.edu/LORE/) or Expedition 378 datasets published in Zenodo (https://publications.iodp.org/proceedings/378/datasets.html).
