# Supplementary material for: Sedimentary hiatus causes abrupt decline and shifts in marine subsurface sediment microbial communities: a study from IODP Exp. 378 Site U1553 offshore southern New Zealand
Source: FEMS Microbiol Lett. 2026 May 14;373:fnag058. doi: 10.1093/femsle/fnag058 (PMC13234949; doi:10.1093/femsle/fnag058)
Supplement: fnag058_Supplemental_Files [file fnag058_supplemental_files.zip › Supplementary_Figure.docx]

**Figure S1. Rarefaction curves for 16S rRNA gene amplicon sequences.** The plot shows the number of observed amplicon sequence variants (ASVs) as a function of the number of reads for each sediment sample. The vertical red dashed line indicates the rarefaction depth (8,550 reads) applied to normalize the sequence data for downstream alpha diversity analysis.

A

B

**Figure S2.** **Relative abundance of microbial communities at the order and family levels.** Bar charts illustrating the taxonomic composition of the microbial communities at Site U1553 at the (A) order and (B) family levels, based on 16S rRNA gene amplicon sequencing. The top 25 most abundant taxa across the samples are displayed individually, while all remaining taxa are grouped together as “Other”.

**Figure S3.** **Principal component analysis (PCA) of the microbial community composition at Site U1553.** The PCA was performed based on Aitchison distance, using centered log-ratio (CLR) transformed amplicon sequence variant (ASV) abundances.

**Figure S4.** **Depth profiles of microbial alpha diversity at Site U1553.** The plots display the Shannon index and the number of observed amplicon sequence variants (ASVs) along the core depth.
